# Health economic impact of early versus delayed treatment of herpes simplex virus encephalitis in the UK

**DOI:** 10.1136/bmjopen-2024-088473

**Published:** 2025-09-18

**Authors:** Sylviane Defres, Patricia Navvuga, Shona Moore, Hayley Hardwick, Ava Easton, Benedict Daniel Michael, Rachel Kneen, Michael Griffiths, ENCEPHUK Study Group, Gavin Barlow, Antonieta Medina-Lara, Tom Solomon

**Affiliations:** 1 Institute of Infection, Veterinary and Ecological Sciences, University of Liverpool, Liverpool, UK; 2 Tropical and Infectious Disease Unit, Liverpool University Hospitals NHS Foundation Trust, Liverpool, UK; 3 Liverpool School of Tropical Medicine, Liverpool, UK; 4 Encephalitis International, Malton, North Yorkshire, UK; 5 The Walton Centre NHS Foundation Trust, Liverpool, Liverpool, UK; 6 Department of Neurology, Alder Hey Children’s NHS Foundation Trust, Liverpool, Merseyside, UK; 7 Health Economics Group, University of Exeter Medical School, Exeter, UK; 8 National Institute for Health Research Health Protection Research Unit in Emerging Zoonotic Infections, University of Liverpool, Liverpool, UK

**Keywords:** HEALTH ECONOMICS, Infectious disease/HIV, Quality of Life, Health economics

## Abstract

**Abstract:**

**Objective:**

Thanks to the introduction of recent national guidelines for treating herpes simplex virus (HSV) encephalitis, health outcomes have improved. This paper evaluates the health system costs and the health-related quality of life implications of these guidelines.

**Design and setting:**

A sub-analysis of data from a prospective, multi-centre, observational cohort ENCEPH-UK study conducted across 29 hospitals in the UK from 2012 to 2015.

**Study participants:**

Data for patients aged ≥16 years with a confirmed HSV encephalitis diagnosis admitted for treatment with aciclovir were collected at discharge, 3 and 12 months.

**Primary and secondary outcome measures:**

Patient health outcomes were measured by the Glasgow outcome score (GOS), modified ranking score (mRS) and the EuroQoL; healthcare costs were estimated per patient at discharge from hospital and at 12 months follow-up. In addition, Quality Adjusted Life Years (QALYs) were calculated from the EQ-5D utility scores. Cost–utility analysis was performed using the NHS and Social Care perspective.

**Results:**

A total of 49 patients were included; 35 were treated within 48 hours, ‘early’ (median (IQR) 8.25 [3.7–20.5]) and 14 were treated after 48 hours ‘delayed’ (median (IQR) 93.9 [66.7–100.1]). At discharge, 30 (86%) in the early treatment group had a good mRS outcome score (0–3) compared with 4 (29%) in the delayed group. According to GOS, 10 (29%) had a good recovery in the early treatment group, but only 1 (7%) in the delayed group. EQ-5D-3L utility value at discharge was significantly higher for early treatment (0.609 vs 0.221, p<0.000). After adjusting for age and symptom duration at admission, early treatment incurred a lower average cost at discharge, £23,086 (95% CI: £15,186 to £30,987) vs £42,405 (95% CI: £25,457 to £59,354) [p<0.04]. A -£20,218 (95% CI: -£52,173 to £11,783) cost difference was observed at the 12- month follow-up post discharge.

**Conclusions:**

This study suggests that early treatment may be associated with better health outcomes and reduced patient healthcare costs, with a potential for savings to the NHS with faster treatment.

**STRENGTHS AND LIMITATIONS OF THIS STUDY**

- Largest cohort of prospectively recruited HSV encephalitis cases in UK to date.
- Systematic 12- month follow-up for clinical economic and quality of life data.
- Small sample disease by virtue of being a rare disease.
- A study was performed from 2012 to 2015, and delays in analysis due to COVID may impact the economic evaluations now.
- Some missing data where patients were transferred from local hospitals.

## Introduction

 Herpes simplex virus (HSV) encephalitis is a rare but severe brain infection, resulting in inflammation and necrosis of the brain parenchyma, which causes significant morbidity and continues to have a mortality of 10%, even when treated with antiviral drugs.^1 2^ In the United Kingdom (UK), approximately 1 in 250 000 to 5 00 000 people are newly diagnosed with HSV encephalitis annually.^3^ Early symptoms of HSV encephalitis include influenza-like symptoms and lethargy, which are also common to a variety of infections while the later symptoms, such as speech problems and seizures, tend to mimic more common brain conditions such as stroke.^4^ The non-specific range of symptoms has been associated with delayed diagnosis and treatment.^5^

A UK study showed that the median time to treatment for suspected HSV encephalitis was 48 (range: 2–432) hours from admission to hospital.^6^ Since the publication of the clinical guidelines for the management of suspected encephalitis in 2012, more recent studies have shown a reduced median time to treatment for suspected encephalitis, around 15 hours.^7 8^ But while a number of studies have reported on the favourable (modified Rankin Scale of 0–3) clinical outcomes associated with the early treatment (≤ 48 hours from admission) of HSV encephalitis, there is limited research into the potential impact on healthcare resource utilisation and costs.^9^,^15^

We sought to evaluate the healthcare cost and health outcome implications of the time interval from hospital admission to HSV encephalitis treatment with aciclovir. We assessed the cost per quality adjusted life year (QALY) and estimated the potential cost savings to the National Health Service (NHS) from improving treatment times in the UK. For the long-term impact, we hypothesise that treating HSV encephalitis patients within 48 hours would result in better neurological outcomes and lower healthcare costs.

## Methods and analysis

### Study design and setting

A sub-analysis was conducted as part of a wider prospective, multi-centre, observational cohort ENCEPH-UK study on ‘Understanding and Improving the Outcome of Encephalitis’ conducted across 29 hospitals in the UK from 2012 to 2015.^16^

### Patient and public involvement statement

The Encephalitis Society, the only patient organisation for encephalitis, was closely involved in the study design and conduct of the study with members from the encephalitis society, and it lay representation from individuals who had experienced encephalitis on our steering committee. The main study has already been disseminated via the encephalitis society and all subsequent studies will do so as well.

### Study participants

This sub-analysis was restricted to patients with confirmed HSV encephalitis aged ≥16 years admitted for treatment with aciclovir 10 mg/kg three times daily (online supplemental Figure 1). Patients were stratified into early treatment for those treated within 48 hours from admission and delayed treatment for those treated ≥48 hours from admission. The 48 hour cut-off was chosen based, as earlier mentioned, on the evidence of significantly better neurological outcomes and health-related quality of life for patients who receive treatment within this time interval from admission compared with patients with delayed treatment.^6 9^

### Data collection

Patient data were collected prospectively using the standardised Case Report Forms (CRF) as part of the wider ENCEPH-UK study.^16^

### Patient demographic and clinical data

Patient demographics – age and gender, clinical data – symptom duration, Charlson comorbidity index, immunocompetent status and Glasgow Coma Score (GCS) severity were recorded on admission. Clinical outcomes including modified Rankin Scale (mRS), where 0–3 represented good outcomes while 4–6 represented poor outcomes, and Glasgow Outcome Score (GOS), on a 1 (death) to 5 (good recovery) scale, were recorded at hospital discharge and at 3 and 12 months follow-up.

### Resource use data and unit costs

Resource use data were collected at hospital discharge and at 3 and 12 months follow-up. This included: (1) length of hospital stay disaggregated into general ward and intensive care unit (ICU) to accurately reflect time spent in the different wards; (2) diagnostic tests [lumbar puncture (LP), electroencephalogram (EEG), MRI and CT]; (3) hospital transfer for those patients that required specialised care and (4) outpatient follow-up costs including follow-up diagnostics, readmission and rehabilitation. Specific unit costs for hospital stay in ICU and general neurological wards were used to accurately reflect resources used in the different wards. The location of clinic for the appointment during follow-up was recorded, which allowed for separate costs to be applied dependent on the location/type of the appointment. Data on each patients’ location at discharge and follow-up time points were recorded, noted either as home, rehabilitation or other. An average cost per episode of neurological rehabilitation for patients with an acquired brain injury was used to derive the cost of rehabilitation. Individual patient resource use data were applied to the corresponding unit costs obtained from the NHS reference costs 2018/19, drugs and pharmaceutical electronic market information tool (eMIT), 2019 where appropriate.^17 18^ All costs are in Great British pounds (GBP£) and a detailed breakdown of the resources utilised and unit costs are available in online supplemental Table 1).

### Health-related quality of life data

Health-related quality of life (HRQoL) data were assessed using the EQ-5D-5L, a five dimension generic preference based health questionnaire that measures patient reported outcomes on (a) mobility, self-care, usual activities, pain/discomfort and depression.^18^ The EQ-5D-5L health states defined by the EQ-5D-5L descriptive system were converted into a single index value using the ‘crosswalk’ between the EQ-5D-3L value sets and the new EQ-5D-5L descriptive system to obtain the index value for the EQ-5D-5L value sets. The EQ-5D utility values at discharge, 3 and 12 months are presented in this analysis. The health economic outcome measure was the QALY, a summary measure of health outcomes that captures the effect of an intervention on both the quality and length of life in a single index unit, comparable across differing diseases.^17 18^ The QALY was generated using the EQ-5D utility values at discharge, 3 and 12 months post discharge.

### Cost utility analysis

We conducted a cost–utility analysis of early treatment compared with delayed treatment of HSV encephalitis with aciclovir on the imputed data. The differences in health outcomes (QALYs) and costs between the two treatment groups at 12 months follow-up were estimated using generalised linear models (GLMs), controlling for baseline covariates. While non-parametric methods of evaluation are typically preferred, results are median estimates, which are not appropriate for the decision maker, who would prefer mean estimates.^19^ A GLM with a Gaussian family and log link was used to predict the mean QALY at 12 months follow-up.^20^

A GLM model was fitted, controlling for age, gender and symptom duration at admission and treatment, to minimise their independent effect on the resource use and costs. A simpler model with only treatment group as a covariate was also fitted; however, model comparison using the Akaike Information Criterion (AIC) and the Bayesian Information Criterion (BIC) showed minimal differences in AIC and BIC estimates (online supplemental Table 2) and as such, the model with age and symptom duration as covariates was selected to adjust for the potential confounding bias.

The mean costs and QALYs were predicted from the GLM model. The incremental cost-effectiveness ratio (ICER) – a ratio of difference in predicted mean costs to difference in predicted mean QALYs – was presented with their confidence intervals. To explore the uncertainty around the ICER, a non-parametric bootstrap technique with 1000 iterations was employed on the mean cost and QALY predictions. Results are presented with the bias-corrected confidence intervals on the mean estimates as well as a cost-effectiveness plane (CEP).

We also projected the estimated average annual savings to the NHS based on annual incidence rate of 0.4 per 100,000,^21^ if HSV encephalitis patients received treatment within 48 hours from admission. We assumed that the proportions of patients at each time to the treatment group were representative of management of HSV encephalitis across the UK to scale up costs to a national level. The analysis was undertaken from the UK NHS perspective and all costs were expressed in pounds (£) for the year 2018/19. All statistical analyses were conducted in Stata/IC 14.1 for Windows.

### Missing data

Missing data were reported in variables – GCS severity, mRS and GOS scores as well as EQ-5D utility scores. Missing values were explored to assess the type of missingness. GCS severity at admission and mRS and GOS at discharge and follow-up contained missing values that were determined to be missing at random, as their probability of being missing was independent of unobserved data. Missingness for each of these variables was between 10% and 12%. Multiple imputation using chained equations (MICE) with a predictive mean matching model was used to impute the missing data on GCS severity, mRS and GOS using 20 imputations. MICE technique allows for the simultaneous imputation of multiple variables while the predictive mean matching model maintains the original distribution structure of the data.^22 23^ The Fraction of Missing Information (FMI) test statistic of 35% validated the choice of 20 imputations as sufficient.

### Statistical analyses

Patient demographics and clinical characteristics at baseline were presented as means±SD, medians with interquartile ranges (IQR) and frequencies with percentages as appropriate. Group differences were assessed using the student’s t-test or Mann–Whitney U test and the χ ^2^ or Fisher’s exact test for continuous and categorical variables, respectively. Group demographic clinical outcome differences were considered significant at p≤0.05.

## Results

### Baseline demographic and clinical characteristics

Of the 341 suspected encephalitis patients recruited, 49 had HSV encephalitis and were stratified into two groups based on the timing of their first dose of aciclovir treatment (table 1). 35 (71%) patients received early while 14 (29%) patients received delayed treatment, at a median of 8.3 [IQR: 3.7 to 20.5] and 93.90 [66.70 to 100.08] hours from admission, respectively. A comparison of the early vs delayed treatment groups revealed significant differences in mean age (54.3 [SD: 16.5] vs 65.8 [SD: 16.3]; p=*0.031*) and median symptom duration before hospital admission of (4.0 [IQR 3–7] vs 1 [IQR 0–5]; p=*0.014*) days, respectively.

**Table 1:** Baseline demographic characteristics and clinical features

Patient characteristics	Early treatment (n=35)	Delayed treatment (n=14)	P value
Age in years, mean (SD)	54.26 (16.46)	65.79 (16.25)	0.031^*^
Gender, n (%) Male	20 (57%)	10 (71%)	0.52^†^
Charlson comorbidity index, n (%) No comorbidity	27 (77%)	8 (57%)	0.18^†^
Immunocompetent status, n (%) Immunocompromised	1 (3%)	1 (7%)	0.49^†^
GCS severity, n (%)			
Severe (≤8) Moderate (9-12) Mild (13-15)	1 (3%) 6 (17%) 28 (80%)	1 (7%) 3 (21%) 10 (71%)	0.58^†^
Symptom duration before admission in days, Median (IQR)	4.00 (3 - 7)	1.00 (1 - 5)	0.014^‡^
Symptom duration to treatment in days, Median [IQR]	5.00 (3–8)	5.00 (5–8)	0.62^‡^
**Clinical outcomes at discharge from hospital**			
**mRS at discharge, n (%)**			
No symptoms at all No significant disability despite symptoms Slight disability Moderate disability Moderate to severe disability Severe disability, bedridden Dead Good (0–3) Poor (4-6)	2 (5.7%) 8 (22.9%) 10 (28.6%) 10 (28.6%) 3 (8.6%) 2 (5.7%) – 30 (86%) 5 (14%)	1 (7.2%) – – 3 (21.4%) 4 (28.6%) 3 (21.4%) 3 (21.4%) 4 (29%) 10 (71%)	0.0002^†^
**GOS score at discharge, n (%)**			
Death Persistent vegetative Severe disability Moderate disability Good recovery	0 (0%) – 14 (40%) 11 (31%) 10 (29%)	3 (21%) – 7 (50%) 3 (21%) 1 (7%)	0.0243^†^
**EQ-5D utility scores at discharge** Median (IQR)	0.61 (0.5–0.8)	0.35 (0–0.4)	<0.0001)^‡^

* Two sample t test used for normally distributed continuous variables

† Fisher’s exact used for categorical variables

‡ Wilcoxon rank-sum used for skewed continuous variables.

GCS, Glasgow Coma Score; GOS, Glasgow Outcome Score.

Significant differences in clinical outcomes at discharge between patient treatment groups were reported for mRS (*p<0.0002*) and GOS score at discharge (*p=0.024*). 71% (10/14) of patients in the delayed treatment group had a poor mRS score at discharge compared with 14% (5/35)) in the early treatment group. Of the 14 patients whose treatment was delayed, 3 died while only 1 patient had good recovery at discharge. 10 of the 35 patients who received treatment within 48 hours of admission had good recovery, with no neurological impairment at discharge. Patients receiving early treatment reported a significantly higher median EQ-5D utility values at discharge compared with delayed treatment patients, 0.61 (0.5–0.8) vs 0.35 (0–0.4): p<*0.0001*.

The results in online supplemental Table 3 below show a significant difference in the median (IQR) length of hospital stay between the early and delayed treatment groups; 31 (22–64) vs 95 (29 – 157) days, respectively, (*p=0.046*). There was no significant difference in the duration of treatment with aciclovir between the two groups (*p=0.13*).

### Unadjusted mean healthcare costs

Results in table 2 below show that the unadjusted mean costs incurred by the patient at discharge were lower for patients receiving early treatment £22,854 (95% CI: £14,180 to £31,528) compared with delayed treatment £42,902 (95% CI: £25,859 to £59,945). The length of stay in hospital was the main driver of initial hospital admission costs, accounting for 91% of all costs incurred by patients treated early and 95% in the delayed treatment group. The mean cost of hospital stay was higher in the delayed treatment group in both the general ward and the ICU.

**Table 2:** Unadjusted Mean costs by treatment group. Results presented as means (95% CI)

Variable	Early Treatment n=35 (95% CI)	Delayed Treatment n=14 (95% CI)	Difference (95% CI)	P value
General ward stay	11 929.1 (9002.8 to 14 855.3)	27 668.6 (16 099 to 39 238.3)	−15,739.6 (-27,565.4 to −3,913.8)	*0.0125*
ICU stay	8939 (-200.18 to 18 078.2)	13 226.1 (2788.4 to 23 663.8)	−4287.1 (-17,684.3 to 9110)	*0.5202*
**Total hospital stay**	20 868.1 **(12 223.6 to** 29 512.5)	40 894.7 **(23 937.1 to** 57 852.3)	**−20,026.7** **(-38,589.8 to −1,463.5**)	*0.0358*
Diagnostic tests (CT, MRI, EEG)	1654.5 (1582.75 to 1726.34)	1597.7 (1497.54 to 1697.9)	56.8 (-62.43 to 176.09)	*0.3377*
Aciclovir antiviral treatment	287.33 (257.05 to 317.6)	317.86 (236.01 to 399.7)	−30.53 (-116.36 to 55.29)	*0.4634*
Ambulance transfer	44.05 (10.29 to 77.82)	91.8 (18 to 165.57)	−47.7 (-127.09 to 31.64)	*0.2237*
**Mean initial admission per patient**	22 853.99 **(14 180.36 to** 31 527.61)	42 902.08 **(25 858.86 to** 59 945.29)	**−20,048.09** **(-1396.8 to** 38 699.4)	*0.0364*
**Variable**	**Early Treatment** **n=33** (**95% CI**)	**Delayed Treatment** **n=10** (**95% CI**)	**Difference** (**95% CI**)	**p-value**
Outpatient clinic visits	501.3 (273.13 to 729.43)	202 (17.63 to 386.17)	−299.4 (18.89 to 579.87)	*0.0371*
Re-admissions at follow-up	655.6 (-427.24 to 1738.39)	1227.6 (-1,382.51 to 3837.71)	−572 (-3315.23 to 2171.18)	*0.6599*
Follow-up diagnostics	104.5 (46.91 to 162.12)	43.4 (-54.78 to 141.58)	61 (-47.99 to 170.22)	*0.2540*
Rehabilitation costs	10 437 (3793.4 to 17 080.7)	25 832 (9927.6 to 41 735.9)	15 394.7 (-1333.1 to 32 122.6)	*0.0683*
Mean follow-up cost per patient	11 698.5 (5126 to 18 270.9)	27 305 (12 411 to 42,198)	15 606.2 (-159.8 to 31 372.4)	*0.0521*
Total average overall costs per patient	34 809.3 (20 658 to 48,960)	71 043 (38 587.1 to 103,498.7)	36 234 (1926.5 to 70 540.8)	*0.0399*

EEG, electroencephalogram; ICU, intensive care unit.

Patients receiving early treatment incurred lower average follow-up costs of £1,216 (95% CI: £172 to £2351) compared with £1,473 (95% CI: £1166 to £4112) for those whose treatment was delayed. Similarly, the average patient cost at 12 months was lower for patients receiving early treatment in comparison to delayed treatment patients, £24,372 (95% CI: £15,007 to £33,738) vs £45,211 (95% CI: £23,014 to 67,409), respectively.

### Adjusted mean healthcare costs

Results show a higher mean patient cost at discharge of £23,086 (95% CI: £15,186 to £30,987) vs £42,405 (95% CI: £25,457 to £59,353) for patients in the early and delayed treatment groups respectively after adjusting for age and symptom duration before admission. This resulted in a difference of -£19,319 (95% C.I: -£37,783 to -£854) (See online supplemental table 2 in Appendix).

Follow-up data were available on patient’ clinical outcomes during the first 12 months post inpatient stay for 43/49 patients. Four of the six patients with data missing on follow-up appointments were in the delayed treatment group. All four individuals had moderate to severe disability at the time of discharge. Three patients died prior to discharge, all of whom were in the delayed treatment group, and a further two during the 12-month follow-up period. Both individuals had been discharged to care homes with more outcome scores and were bedridden. After adjusting for age and symptom duration before admission, there was a difference of -£6,570 (95% CI: - £25,326 to £12,185) in mean patient follow-up costs. The average costs from admission to 12 months post-discharge for patients on early treatment were £38,359 (95% CI: 22 470 to £54,247) vs £58,576 (95% CI: 32 141 to £85,011) for those receiving delayed treatment, resulting in a difference of -£20,217 (95% CI: -£52,173 to £11,738).

### Cost-utility analysis

Table 4 presents a summary of the bootstrapped estimates for mean costs and QALYs for patients in the early and delayed treatment groups. The average cost per patient was higher in the delayed treatment group [£76,071 (BC 95% CI: £58,037 to £105,743) compared with the early treatment group [£34,866 (BC 95% CI: £30,715 to £39,423)]. Similarly, patients receiving early treatment reported better health outcomes with a mean QALY at 12 months of 0.613 (BC 95% CI: 0.599 to 0.630) compared with patients receiving delayed treatment 0.492 (BC 95% CI: 0.474 to 0.515).

**Table 3:** Bootstrapped adjusted mean costs, QALYs and ICER

Treatment group	Mean total costs (BC 95% CI)	Mean QALYs (BC 95% CI)	Incremental cost (95% CI)	Incremental QALY (95% CI)
**Early Treatment**	£34,886 (£30,715 - £39,423)	0.613 (0.599 to 0.630)	-£41,185 (-£40,891 to -£38,815)	0.125 (0.121 to 0.123)
**Delayed Treatment**	£76,071 (£58,037 - £105,743)	0.488 (0.464 to 0.522)		

ICER, incremental cost effectiveness ratio; QALY, quality adjusted life years.

**Figure 1:**
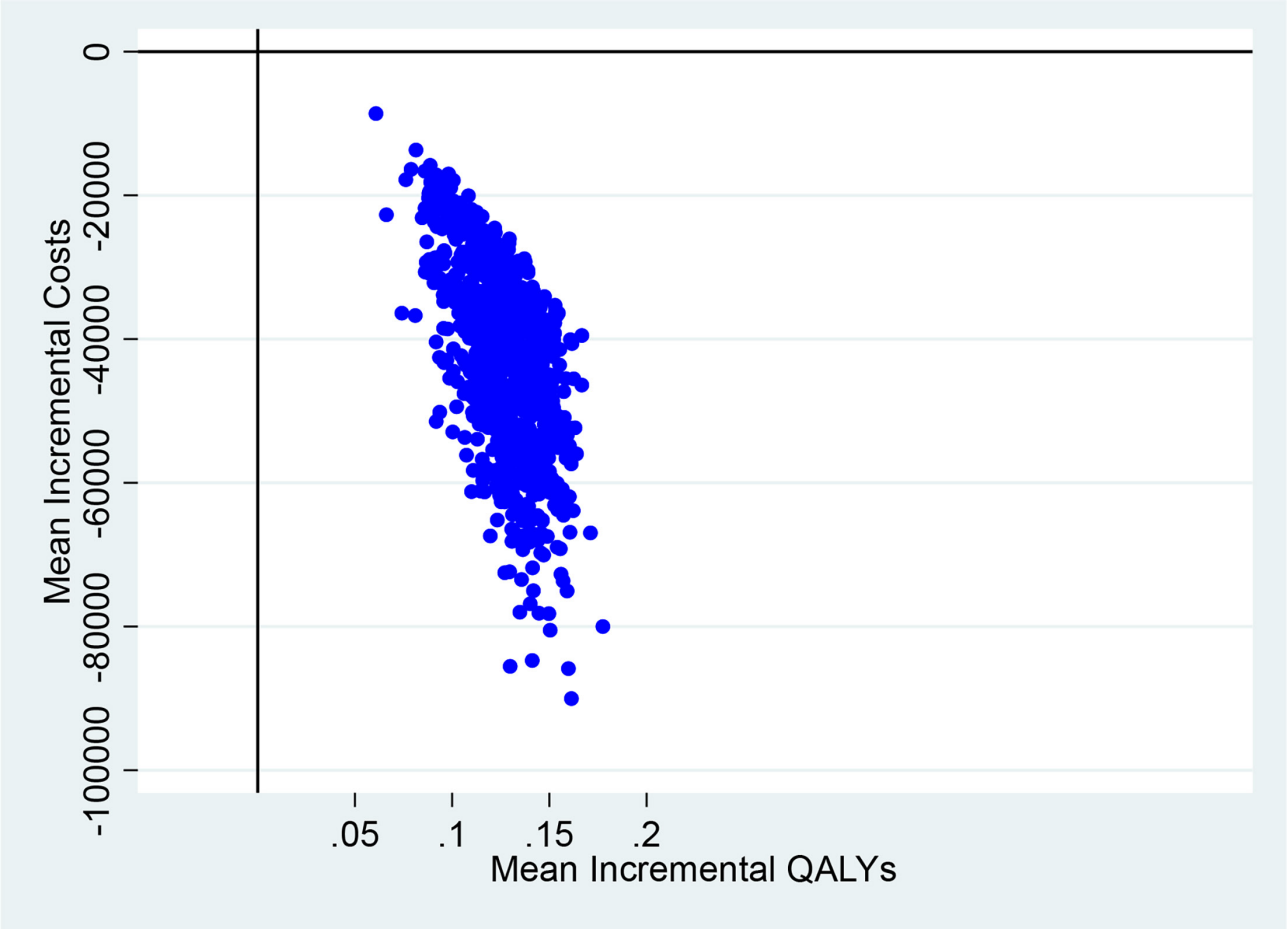
Cost Effectiveness Plane

Figure 1 is the CEP, which shows that early treatment is both less costly and more effective and therefore dominates receiving delayed treatment.

## Discussion

### Principle findings from the Study

HSV encephalitis can have devastating long-term consequences for patients, with many experiencing neurological impairments^15^ and on-going health issues, such as memory problems, epilepsy and behavioural changes.^24^ Several studies have reported on the association between time to treatment with aciclovir and the clinical outcomes of patients with HSV encephalitis.^12 15^ These clinical outcomes are often measured using the Glasgow Outcome Scale (GOS) or modified Rankin Scale (mRS), and a number of studies found a significant association between delayed treatment with aciclovir and poor health outcomes, with the majority of studies classifying delay as greater than either one or 2 days.^9^,^1425^ Although these studies have differed in the settings, populations and some of the outcome measures they used, they all showed that there is an increased chance of poor outcome with a longer time to treatment. Findings of these studies are in alignment with our analysis which found that 71% of patients in the delayed treatment groups reported a ‘poor’ mRS score at discharge compared with only 14% in the early treatment group (significant at p<0.0002). Similarly, for the GOS score at discharge, good recovery was reported of only 7% of the patients whose treatment was delayed compared with 29% of patients being treated early. However, these outcome measurement tools are crude and not nuanced and detailed enough to demonstrate more subtle problems that may impact on activities of daily living and employment, eg, lack of concentration, poor memory and fatigue.

No previous study has looked at the effect of treatment delays on healthcare resource, costs and HRQoL, as well as estimating cost effectiveness of early treatment. After adjusting for confounding, the mean cost at 12 months follow-up was £34,886 (BC 95% CI: £30,715 to £39,423) and £76,071 (BC 95% CI: £58,037 to £105,743) for patients receiving early and delayed treatment, respectively. It is important to note that the length of hospital stay, the main driver of healthcare costs incurred by the patients, was somewhat significantly longer for patients in the delayed treatment group (94 [IQR: 29–157] days) compared with the early treatment group (31 [IQR: 22–58] days), (*p<0.05*). The resulting difference in the overall mean healthcare costs between the two treatment groups was - £39,960 (BC 95% CI: -£40,891 to -£38,815). QALYs were higher in the early treatment group compared with the delayed treatment group, 0.613 (BC 95% CI: 0.599 to 0.630) and 0.488 (BC 95% CI: 0.464 to 0.522), respectively. This resulted in a cost per QALY of -£334,068 (-£339,523 to -£329,856). However, statistical significance should be interpreted with caution due to the lack of power in the analysis associated with the small sample size as well as the potential selection bias which occurs when the differences in demographic and clinical characteristics prior to treatment have an independent effect on the patient outcomes.^26^ These estimated healthcare costs are likely to be an underestimation of the true costs of treating HSV encephalits,as previous studies have shown patients often suffer from long-term sequelae many years after initial presentation.^27 28^ Therefore, it is probable that additional costs to the NHS in terms of GP attendances, further diagnostic investigations and costs associated with ongoing care may have been under-reported in the study. In addition, an earlier study by our group found that the sequelae after HSV encephalitis had significant impacts on activities of daily living with patients likely to incur costs through productivity losses due to difficulties returning to work either in their prior or in a changed role; there are additional costs for family/friends who become carers.^24^ However, given that the perspective of our analysis was that of the payer (NHS), these wider social costs were not included in the analysis.

We also projected the potential annual cost to the NHS of treating HSV encephalitis patients in the UK by assuming that the proportions of patients in each time to treatment group are representative of management of HSV encephalitis across the UK. Scaling up costs to a national level equated to average healthcare costs of £11.7 million annually, based on an annual incidence rate of 0.4 per 100,000.^21^

The differences in average healthcare costs between those treated more promptly and those delayed highlight the potential for savings for the NHS through reducing treatment delays. Significant improvements have already taken place in the UK regarding management of HSV encephalitis over the past 20 years. Our group led the development and publication of guidelines for the management of suspected viral encephalitis, facilitating improvements in recognition and diagnosis of the condition to reduce delays in treatment.^21 29^ Without the availability of time-series data following the same hospitals over time, it is difficult to measure the precise extent of any improvements. However, the earliest UK study reported a median time to treatment of 48 hours, with 56% of patients experiencing delays of 48 hours or more compared with 29% in this recent study.^6^ This is still too high. The national guidelines suggest all patients should be on treatment within 6 hours of hospital admission. National level costs of a case of HSV encephalitis preguideline publication can be estimated by assuming the proportions of patients treated early vs delayed in the earliest study^6^ are representative of UK management during that time (44% treated early, 56% delayed) and applying the average costs for each treatment group estimated from this current study, adjusted for inflation using the consumer price index with 2015 as the base year.^30^ Based on average costs estimated in our study, this would have equated to past (pre-guidelines) annual healthcare costs of £11.7 million in the UK. If the reduction in the proportion of patients experiencing delays through improvements in management over time is reflective of the UK population as a whole, this would equate to an annual saving of £720,000.

Symptoms of HSV encephalitis, particularly in the early stages of disease, have similarities with many other viral infections. However, the disease can rapidly develop over days, making prompt treatment with aciclovir crucial. There are a number of reasons that could result in treatment delays, which were not explored in this current study. Previous studies have shown that delays in undertaking CT scans can result in delays in the initiation of aciclovir,^6 11^ as can a failure to recognise HSV encephalitis as an initial diagnosis, due to the non-specific nature of the illness.^31^ This was also highlighted in a study conducted with HSV encephalitis survivors and their families, who described the difficulties in navigating healthcare systems during their illness trajectories, often having to develop their own care pathways in order to obtain recognition for their symptoms/ illness.^3^ The study emphasised the importance of involving and listening to patients and their significant others concerning symptom recognition and changes in usual behaviour to assist with the diagnosis and reduce treatment delays. In our current study, there were significant differences in the duration of symptoms prior to hospital admission between the early and late treatment groups, and it is possible that those with longer durations of symptoms may have taken their illness more seriously, whereas those with shorter durations of symptoms may have been observed prior to investigating. Also, there were significant differences in age between the two groups were observed (*p=0.031*), with those with delayed treatment being older on average. Differences in age may influence whether a patient is treated/diagnosed quicker for a number of reasons. For example, it may be more difficult to recognise symptoms of encephalitis in older patients, such as confusion or dizziness, and often their symptoms are attributed to other conditions common in the elderly such as delirium or stroke. Discharging elderly patients is also more complex thus contributing to the length of stay, which in this study was demonstrated to be the major driver of the cost difference between the two groups. Previous literature has also shown older age to be associated with poor outcome.^12 25^ The observation that those delayed were older on average highlights that further improvements are needed on the management of suspected encephalitis in elderly patients, with those already at an increased chance of poor outcome being further disadvantaged through treatment delays.

### Limitations of the analysis

There are a number of limitations to this study. First, as a result of the rarity of HSV encephalitis and missing data on some patients’ time of admission due to patients being transferred from other hospitals, this was a statistically small sample size. A small sample size could potentially underpower the analysis and result in inadequate treatment effect estimates.^32^ There is also a risk that the results could be biased in terms of differences in the characteristics of those individuals where missing data around the admission and administration of aciclovir times was concerned. While 16 individuals’ data were not included due to this missingness, looking at their characteristics and outcomes does not suggest any bias with their data that is missing being randomly distributed. Second, as is usually the case with non-randomised studies, selection bias, which was not adequately identified and adjusted for in this analysis, meant that the unadjusted comparison of mean cost estimates are biased and should be interpreted with extreme caution as well as the extrapolations. Attempting to correct for selection bias with a matching technique would lead to an even smaller sample size and the potential loss of informative data points.^26^ Third, relying on case report forms to record patients resource use to estimate healthcare costs may not capture all the costs incurred. This is particularly true for follow-up data where primary care and community care contact outside the study hospitals will not have been captured. In addition, as is common during studies with follow-up periods, some patients had missing data in the 3- and 12-month CRFs, further reducing the sample size. There were a number of reasons for this missing data, including withdrawal due to the burden of follow-up, particularly when experiencing significant sequelae, and returning to relatives’ homes instead of their own address due to need for carers. Follow-up costs were only captured for the first 12 months post discharge and did not include primary and community care costs, which are likely to be substantial for those HSV encephalitis patients, particularly with recent studies showing the encephalitis patients often suffer from ongoing sequelae many years after initial presentation.^24 27 28^ An additional limitation is the imbalance in the follow-up of patients between the early and delayed treatment groups. The final limitation is that this study is regarding when the initial data was collected from. Delays in data cleaning and the subsequent SARS-CoV pandemic had an impact on the analysis of the study. As such calculations are based on costs at that time, which will be different to current day charges and the cost-of-living crisis will have had an impact on out-of-pocket costs to patients and families. Future studies should examine differences in time to treatment delays on longer term follow-up costs.

## Conclusions and implications

HSV encephalitis can have devastating consequences for patients. This study has shown that those treated more promptly had significantly lower inpatient stay costs than those with delayed aciclovir treatment. This was also seen during the first year after discharge. Alongside previous literature showing that delays in treatment result in poorer patient outcomes, the results of this study show that there are also significant costs to the NHS as a consequence of these delays. Work has been undertaken in the UK over recent years to improve the management of suspected encephalitis and encourage prompt treatment with aciclovir if the condition is suspected, which has led to reductions in average treatment times. Based on the findings of this study, reducing the number of patients experiencing delays and thus improving the management of HSV encephalitis is likely to have resulted in savings to the NHS. In addition, this study indicates the potential savings that could be made in the future with further improvements in the management of HSV encephalitis in the UK. Results of this analysis validate the importance of the encephalitis management guidelines that have boosted efforts towards timely diagnosis and instigation of treatment.

## Supplementary material

10.1136/bmjopen-2024-088473 online supplemental file 1

## Data Availability

All data relevant to the study are included in the article or uploaded as supplementary information.
