# Supplementary material for: Health economic impact of early versus delayed treatment of herpes simplex virus encephalitis in the UK
Source: BMJ Open. 2025 Sep 18;15(9):e088473. doi: 10.1136/bmjopen-2024-088473 (PMC12458707; doi:10.1136/bmjopen-2024-088473)
Supplement: online supplemental file 1 [file bmjopen-15-9-s001.docx]

**Supplemental Table 1: Resource Unit Costs**

|  | **Resource Use** | **Unit cost** | **Description** | **Source** |
| --- | --- | --- | --- | --- |
| **Imaging and Diagnostics** | | | |  |
|  | MRI | £183 | Diagnostic imaging (Direct access): RD03Z- Magnetic Resonance Imaging Scan of one area, with pre and post contrast. | NHS Reference Costs 2018-19 |
|  | CT scan | £138 | Diagnostic imaging (Direct access): RD22Z- Computerised Tomography Scan of one area, with pre and post contrast. | NHS Reference Costs 2018-19 |
|  | EEG | £76 | Directly Accessed diagnostic services: Conventional EEG, EMG or Nerve Conduction Studies, 19 years and over | NHS Reference Costs 2018-19 |
| **Inpatient stay** | | | |  |
|  | General bed day | £317 | AA22C-AA22G: Cerebrovascular Accident, Nervous System Infections or Encephalopathy- Weighted average non-elective inpatient excess bed days | NHS Reference Costs 2018-19 |
|  | ICU bed day | £1,041 | Adult Critical care-Neurosciences adult patients: weighted average of XC05Z-XC07Z, 0-2 organs supported | NHS Reference Costs 2018-19 |
|  | Aciclovir | £10.68 | Aciclovir 500mg/20ml solution for infusion vials / Packsize 5 | Drugs and pharmaceutical electronic market information tool (eMIT)- Department of Health and Social Care |
|  |  | £2.14 | Per vial of 500mg |  |
|  | Ambulance transfer | £247 | Ambulance: ASS02- See and treat and convey | NHS Reference Costs 2018-19 |
| **Clinic/outpatient follow up** | | | | |
|  | Hospital general clinic | £152 | Total Outpatient Attendances-service code 300 | NHS reference costs 2018-19 |
|  | Neurology clinic | £168 | Total Outpatient Attendances-service code 400 | NHS reference costs 2018-19 |
|  | Infectious diseases clinic | £234 | Total Outpatient Attendances-service code 350 | NHS reference costs 2018-19 |
|  | Physiotherapy | £49 | Total Outpatient Attendances-service code 650 | NHS reference costs 2018-19 |
|  | Occupational therapy | £65 | Total Outpatient Attendances-service code 651 | NHS reference costs 2018-19 |
|  | Speech and language therapy | £97 | Total Outpatient Attendances-service code 652 | NHS reference costs 2018-19 |
|  | Psychiatry | £85 | Total Outpatient Attendances-service code 722 | NHS reference costs 2018-19 |
|  | Neuropsychology | £168 | Total Outpatient Attendances-service code 400 | NHS reference costs 2018-19 |
|  | Neuro Rehabilitation | £220 | Rehabilitation- weighted average of rehabilitation services for brain injuries and other neurological disorders levels 1-3 (service description:Other) VC60Z and VC12Z | NHS reference costs 2018-19 |
|  | Rehabilitation | £162 | Total Outpatient Attendances-service code 314 | NHS reference costs 2018-19 |
|  | Clinical phycology | £169 | Total Outpatient Attendances-service code 656 | NHS reference costs 2018-19 |
|  | Transient ischaemic attack (TIA) | £174 | Total Outpatient Attendances-service code 329 | NHS reference costs 2018-19 |
|  | Neuro community rehabilitation team | £92 | Community health services -NCRT: Neuro community rehabilitation teams | NHS reference costs 2018-19 |
| **On-going rehabilitation** | | | | |
|  | Inpatient rehab episode | £43,053 | Average episode cost of specialist inpatient neurorehabilitation stay following acquired brain injury | Turner-Stokes et al. 2016. {Turner-Stokes, 2015 #14} |

Footnote 1: NHS - National Health Service; EMIT – Electronic Marketing Information Tool; BNF – British National Formulary PSSRU – Personal Social Services Research Unit - Unit Costs for Health and Social Care

**Supplemental Figure 1**: Flowchart of patient inclusion from the ENCEPH-UK prospective cohort study

**Supplemental Table 2: AIC and BIC model statistics**

|  | Model | AIC | BIC |
| --- | --- | --- | --- |
| **QALYs** | GLM: log link, gamma family  Covariates: treatment, age, gender, symptom duration to admission/treatment | -16 | -5.5 |
|  | GLM: log link, gamma family  Covariates: treatment | -20 | -17 |
| **Costs** | GLM: log link, gamma family  Covariates: treatment, age, gender, symptom duration to admission/treatment | 1004 | 1013 |
|  | GLM: log link, gamma family  Covariates: treatment | 1003 | 1007 |

**Supplemental Table 3 Patient resource use at discharge from hospital**

| **Patient resources** | **Early treatment (n=35)** | **Delayed treatment (n=14)** | ***p*-value** |
| --- | --- | --- | --- |
| **Hospital stay in days, Median [IQR]** | 31 (22 - 64) | 95 (29 - 157) | 0.0463^c^ |
| **Days on Aciclovir,** Mean (SD) | 21 (8.24) | 25 (9.07) | 0.13^a^ |

^a^Two sample t test, ^b^Fisher's exact, ^c^Wilcoxon rank-sum.
