# Supplementary material for: Health economic impact of early versus delayed treatment of herpes simplex virus encephalitis in the UK
Source: BMJ Open. 2025 Sep 18;15(9):e088473. doi: 10.1136/bmjopen-2024-088473 (PMC12458707; doi:10.1136/bmjopen-2024-088473)
Supplement: Supplementary file 2 [file bmjopen-2024-088473.reviewer_comments.pdf]

PEER REVIEW HISTORY

BMJ Open publishes all reviews undertaken for accepted manuscripts. Reviewers are asked to complete a checklist review form and are provided with free text boxes to elaborate on their assessment. These free text comments are reproduced below.

ARTICLE DETAILS

Title (Provisional)

Health economic impact of early versus delayed treatment of herpes simplex virus encephalitis in the UK

Authors

Defres, Sylviane; Navvuga, Patricia; Moore, Shona; Hardwick, Hayley; Easton, Ava; Michael, Benedict Daniel; Kneen, Rachel; Griffiths, Michael; Study Group, ENCEPHUK; Medina-Lara, Antonieta; Solomon, Tom

---

VERSION 1 - REVIEW

**Reviewer**	**1**
**Name**	**Fillatre, Pierre**
**Affiliation**	**Centre Hospitalier Yves-Le Foll**
**Date**	**25-Nov-2024**
**COI**	**None**

In this manuscript, the authors aimed to estimate the healthcare costs associated with delayed aciclovir treatment in HSV encephalitis. This is an ancillary study of the recently published ENCEPH-UK cohort, which focuses on patients with confirmed HSV encephalitis. Patients were divided based on whether they received treatment within 48 hours or more than 48 hours after admission. Healthcare costs were estimated and compared between the two groups.

The manuscript is interesting as it reinforces the importance of early treatment from a less commonly described perspective (healthcare costs). The methodology is appropriate, and despite the small sample size, the results are significant. I believe that the readers of BMJ Open will be interested in this manuscript. However, I have a few questions and suggestions.

Introduction:

- I believe the authors are referring to HSV-1 throughout the manuscript, although only HSV is mentioned (which implies both HSV-1 and HSV-2). I suggest replacing HSV with HSV-1 throughout the manuscript.

- There is also necrosis, not just inflammation, which likely contributes to the severity of the condition.

- References 7 and 16 seem similar?; please verify.

- Second paragraph, first sentence: 48 hours : from admission or from onset?

Methods:

- Sixteen patients (25%) are missing (supplementary figure 1) due to missing data on the timing of aciclovir initiation. What was their outcome and healthcare cost? This could introduce a selection bias. If these missing data were not randomly distributed, it raises questions. This should at least be more discussed in the limitations section.

- Even though multiple imputations are adequate and the tests used are correct, can the authors provide the number of missing data points? The authors mention that missing data were reported in variables such as GCS severity, mRS, and GOS scores but I did not find it.

- The authors have very interesting and detailed data, particularly regarding the timing of aciclovir administration, which is recorded in hours, whereas most papers only have data in days. Regarding the distribution of timing: how many patients fall within the [0-6] hours, [6-48] hours, and >48 hours ? Would it be possible to further divide the first group (<48h) to evaluate the cost and clinical prognosis of patients receiving aciclovir within the recommended <6h delay after admission and those with a delay of 6-48h? The median for the first group (n=35) is 8h, so there might be around fifteen patients ≤6h, then there may be three equivalent groups?

Results:

- It is surprising to see that the median duration of symptoms before treatment was 4 days in the early treatment group and 1 day in the late treatment group. Thus, the treatment delay is not different concerning symptom onset, which is surprising. Authors may have further discussion in the manuscript on it.

- It would also be very interesting to have data in table 1 on lumbar puncture (cellularity, protein levels), imaging (abnormalities? and if available, the number of lobes affected), and EEG findings.

- To simplify Table 1, I would suggest consolidating some variables: gender, Charlson score, and immunocompromised status could each take up only one line : gender (male, n (%)) or only (female, n(%))...

- I strongly believe that details on the mRS should be included in Table 1, just as the authors have done for the GOS

- Sometimes aciclovir is spelled with an "i" in the text and sometimes with a "y" (as in Table 2); please standardize the spelling.

- To simplify the manuscript, I am not sure it is necessary to discuss both models: adjustment for age and symptom duration before treatment, and adjustment for treatment alone because: 1- for QALYs, AIC and BIC are lower in the first model, so this model is more accurate. 2- there is a difference in the age distribution of patients in the early and late treatment groups (more than 10 years).

- Perhaps the cost differences are related to the fact that it is more challenging to discharge an elderly patient to home than a younger patient. Moreover, the authors have demonstrated that the difference in hospitalization costs is mainly due to the length of stay in acute care.The authors should consider discussing this point in the discussion section.

**Reviewer**	**2**
**Name**	**Phang Romero Casas, Carmen**
**Affiliation**	**Fundacao Oswaldo Cruz, Center for Health Technology in Health**
**Date**	**18-Apr-2025**
**COI**	**None**

This paper evaluates the costs and the health-related quality of life implications of recent national guidelines for treating herpes simplex virus (HSV) encephalitis in UK.

The manuscript is well written, the methodology is enough detailed to be replicated, and the analysis of results are clear.

I recommend the publication of the manuscript, even though just few comments deserve to be made.

ABSTRACT

Between the results of outcomes assessed did not mention Glasgow Outcome Score (GOS) measure results. Cost results presented in what perspective? Since the authors pointed out that cost-utility was performed using the NHS and Social Scare perspectives.

The conclusion also did not mention the social care perspective.

METHODS AND ANALYSIS

Either Resource use data and unit costs and Health-related quality of life (HRQoL) data figured out measure at 3 months. What was the reason for assessing this point? Have any results to show?

RESULTS

Follow-up data during the first 12 months showed an imbalance in losses between groups. Were the losses related to delayed treatment? Were these deaths investigated?

Pag. 14/28

The results at the end of first paragraph “patients receiving delayed treatment 0.492 (BC 95% CI: 0.474 - 0.515)” are different to those presented in Table 4: Mean QALYs (BC 95% CI): 0.488 (0.464 - 0.522). Could you explain this?

LIMITATIONS

Would the authors consider the imbalance in losses between groups (early and delayed treatment) as an additional limitation?

OTHERS

Social Scare perspective is only mentioned in the abstract and no results or analysis were presented for this perspective.

VERSION 1 - AUTHOR RESPONSE

Reviewer: 1
Introduction:
- I believe the authors are referring to HSV-1 throughout the manuscript, although only HSV is mentioned (which implies both HSV-1 and HSV-2). I suggest replacing HSV with HSV-1 throughout the manuscript.

Response

We have left it as HSV as although the majority of our cases of HSV encephalitis were indeed due to HSV type 1, 2 individuals had proven HSV type 2 with confirmed evidence of encephalitis.

- There is also necrosis, not just inflammation, which likely contributes to the severity of the condition.

Response

We totally agree this is the case and have added this to the text.

- References 7 and 16 seem similar?; please verify.

Response

Thank you for spotting that error. Reference 7 in fact was meant to be a different study and this has now been amended.

- Second paragraph, first sentence: 48 hours : from admission or from onset?

Response

This has been clarified in the text to state from hospital admission.

Methods:
- Sixteen patients (25%) are missing (supplementary figure 1) due to missing data on the timing of aciclovir initiation. What was their outcome and healthcare cost? This could introduce a selection bias. If

these missing data were not randomly distributed, it raises questions. This should at least be more discussed in the limitations section.

Response

Thank you for this comment. Yes, indeed these sixteen patients had missing data on their aciclovir prescriptions. Yes, the missing data were randomly distributed and I have added a comment in the limitations section regarding this.

- Even though multiple imputations are adequate and the tests used are correct, can the authors provide the number of missing data points? The authors mention that missing data were reported in variables such as GCS severity, mRS, and GOS scores but I did not find it.

Response

Our apologies for our error, the missingness was for mRS and GOS scores, not for GCS severity. We have added into the text of the manuscript the missingness of the mRS and GOS scores which was then imputed for further analysis.

- The authors have very interesting and detailed data, particularly regarding the timing of aciclovir administration, which is recorded in hours, whereas most papers only have data in days. Regarding the distribution of timing: how many patients fall within the [0-6] hours, [6-48] hours, and >48 hours ?

Response

This is an excellent question. We do have this additional information and have included it in the additional information in the appendix

Would it be possible to further divide the first group (<48h) to evaluate the cost and clinical prognosis of patients receiving aciclovir within the recommended <6h delay after admission and those with a delay of 6-48h? The median for the first group (n=35) is 8h, so there might be around fifteen patients ≤6h, then there may be three equivalent groups?

Response

This is an interesting idea, and we thank the reviewer for this good suggestion. Because there is only a small number of patients in this <6 group (14 patients) and because of the risks of bias with too many post hoc analyses, we have decided not to perform this analysis, because we feel it would be hard to make a sensible interpretation of the result.

Results:
- It is surprising to see that the median duration of symptoms before treatment was 4 days in the early treatment group and 1 day in the late treatment group. Thus, the treatment delay is not different concerning symptom onset, which is surprising. Authors may have further discussion in the manuscript on it.

Response
Yes, this is an interesting observation that we have considered this further in the discussion section of the manuscript.

- It would also be very interesting to have data in table 1 on lumbar puncture (cellularity, protein levels), imaging (abnormalities? and if available, the number of lobes affected), and EEG findings.

Response

Thank you for this comment, we had reported all of this data in the main manuscript of the ENCEPH UK cohort study and for this paper we focused on the health economic analyses.

- To simplify Table 1, I would suggest consolidating some variables: gender, Charlson score, and immunocompromised status could each take up only one line : gender (male, n (%)) or only (female, n(%))...

Response

Thank you for this helpful suggestion, I have amended this in the manuscript

- I strongly believe that details on the mRS should be included in Table 1, just as the authors have done for the GOS

Response

Thank you for this comment. I have changed the binary outcomes of mRS to the itemised results to be in keeping with the GOS scores as recommended.

- Sometimes aciclovir is spelled with an "i" in the text and sometimes with a "y" (as in Table 2); please standardize the spelling.

Response

Thank you for spotting the two times this was missed in the table. I have corrected these. I have left acyclovir in the references as that is how they were published. I trust this is ok.

- To simplify the manuscript, I am not sure it is necessary to discuss both models: adjustment for age and symptom duration before treatment, and adjustment for treatment alone because: 1- for QALYs, AIC and BIC are lower in the first model, so this model is more accurate. 2- there is a difference in the age distribution of patients in the early and late treatment groups (more than 10 years).

Response

Thank you for this comment. I have kept the 1st model has suggested and moved the second model to the appendix for further reading.

- Perhaps the cost differences are related to the fact that it is more challenging to discharge an elderly patient to home than a younger patient. Moreover, the authors have demonstrated that the difference in hospitalization costs is mainly due to the length of stay in acute care. The authors should consider discussing this point in the discussion section.

Response

Indeed this is very true and so we have brought this detail out in more detail in the discussion sections

Reviewer: 2
Dr. Carmen Phang Romero Casas, Fundacao Oswaldo Cruz
Comments to the Author:
This paper evaluates the costs and the health-related quality of life implications of recent national guidelines for treating herpes simplex virus (HSV) encephalitis in UK.
The manuscript is well written, the methodology is enough detailed to be replicated, and the analysis of results are clear.

I recommend the publication of the manuscript, even though just few comments deserve to be made.
ABSTRACT
Between the results of outcomes assessed did not mention Glasgow Outcome Score (GOS) measure results. Cost results presented in what perspective? Since the authors pointed out that cost-utility was performed using the NHS and Social Scare perspectives.
The conclusion also did not mention the social care perspective.

Response
Thank you for your suggestions regarding the abstract. We have amended the abstract to include the Glasgow outcome Score. We have clarified that the cost analyses are from the healthcare system perspective. The reference document used for such costs is called the NHS and Social Care perspectives; however, the focus of this study was only on the NHS healthcare cost, not the social care or out-of-pocket costs.

METHODS AND ANALYSIS
Either Resource use data and unit costs and Health-related quality of life (HRQoL) data figured out measure at 3 months. What was the reason for assessing this point? Have any results to show?

Response
Thank you for raising this question. Although multiple time points were suggested by the health economist, clinical experience shows that patients often worsen initially before then improving. Therefore, 12-month outcomes were used as the most clinically meaningful outcome and with the largest available data set.

RESULTS
Follow-up data during the first 12 months showed an imbalance in losses between groups. Were the losses related to delayed treatment? Were these deaths investigated?
Response
Thank you for your observation. Yes, indeed there were 6 lost to follow-up of which 4 were in the delayed treatment group compared to 2 in the early treatment group. All 4 of these individuals had moderate to severe outcomes at discharge. These individuals are likely to have gone to live with family or go to nursing homes out with catchment areas for research teams to follow-up on, which we find often in clinical practice. The additional deaths were in individuals who had severe disabilities on discharge and went to care homes and it is likely these were related to their HSV encephalitis. We have added some additional comments to the manuscript to explain.

Pag. 14/28
The results at the end of first paragraph "patients receiving delayed treatment 0.492 (BC 95% CI: 0.474 - 0.515)" are different to those presented in Table 4: Mean QALYs (BC 95% CI): 0.488 (0.464 - 0.522). Could you explain this?

Response
Thank you for highlighting this. It was not clearly explained that these were 2 models with slightly different models of adjustment. For clarity we have removed the second model to the appendix and explained that the 1st model is adjusted for age and symptom duration whereas the 2nd was adjusted for treatment alone.

LIMITATIONS
Would the authors consider the imbalance in losses between groups (early and delayed treatment) as an additional limitation?

Response

Thank you for highlighting this imbalance. We have added this as an additional limitation.

OTHERS
Social Scare perspective is only mentioned in the abstract and no results or analysis were presented for this perspective.

Response

As discussed in the 1st point, the study analyses the NHS healthcare perspective not the social costs. It is the name of the reference document for cost calculations that happens to include both in their document. However, the manuscript analyses alone healthcare costs, no social care analyses were conducted.

VERSION 2 - REVIEW

**Reviewer**	**1**
**Name**	**Fillatre, Pierre**
**Affiliation**	**Centre Hospitalier Yves-Le Foll**
**Date**	**27-Aug-2025**
**COI**	

I have carefully read the revised manuscript. All the suggestions I have made before were taken into account. The manuscript has been considerably improved and I believe it is now suitable for publication. The authors should be congratulated for this.

**Reviewer**	**2**
**Name**	**Phang Romero Casas, Carmen**
**Affiliation**	**Fundacao Oswaldo Cruz, Center for Health Technology in Health**
**Date**	**12-Aug-2025**
**COI**	

I agree that the authors addressed all the questions and comments of reviewers and I´m satisfied with the final version of the manuscript.
